# EasyCluster2: an improved tool for clustering and assembling long transcriptome reads

**DOI:** 10.1186/1471-2105-15-S15-S7

**Published:** 2014-12-03

**Authors:** Vitoantonio Bevilacqua, Nicola Pietroleonardo, Ely Ignazio Giannino, Fabio Stroppa, Domenico Simone, Graziano Pesole, Ernesto Picardi

**Affiliations:** 1 Department of Electrical and Information Engineering, Polytechnic of Bari, 70125 Bari, Italy; 2 Department of Biosciences, University of Milan, 20133 Milan, Italy; 3 Department of Biosciences, Biotechnology and Biopharmaceutics, University of Bari, 70126 Bari, Italy; 4 Institute of Biomembranes and Bioenergetics, National Research Council, 70126 Bari, Italy; 5 National Institute of Biostructures and Biosystems (INBB), 00136 Roma, Italy; 6 Center of Excellence in Comparative Genomics, University of Bari, 70126 Bari, Italy

## Abstract

**Background:**

Expressed sequences (e.g. ESTs) are a strong source of evidence to improve gene structures and predict reliable alternative splicing events. When a genome assembly is available, ESTs are suitable to generate gene-oriented clusters through the well-established EasyCluster software. Nowadays, EST-like sequences can be massively produced using Next Generation Sequencing (NGS) technologies. In order to handle genome-scale transcriptome data, we present here EasyCluster2, a reimplementation of EasyCluster able to speed up the creation of gene-oriented clusters and facilitate downstream analyses as the assembly of full-length transcripts and the detection of splicing isoforms.

**Results:**

EasyCluster2 has been developed to facilitate the genome-based clustering of EST-like sequences generated through the NGS 454 technology. Reads mapped onto the reference genome can be uploaded using the standard GFF3 file format. Alignment parsing is initially performed to produce a first collection of pseudo-clusters by grouping reads according to the overlap of their genomic coordinates on the same strand. EasyCluster2 then refines read grouping by including in each cluster only reads sharing at least one splice site and optionally performs a Smith-Waterman alignment in the region surrounding splice sites in order to correct for potential alignment errors. In addition, EasyCluster2 can include unspliced reads, which generally account for *>*50% of 454 datasets, and collapses overlapping clusters. Finally, EasyCluster2 can assemble full-length transcripts using a Directed-Acyclic-Graph-based strategy, simplifying the identification of alternative splicing isoforms, thanks also to the implementation of the widespread AStalavista methodology. Accuracy and performances have been tested on real as well as simulated datasets.

**Conclusions:**

EasyCluster2 represents a unique tool to cluster and assemble transcriptome reads produced with 454 technology, as well as ESTs and full-length transcripts. The clustering procedure is enhanced with the employment of genome annotations and unspliced reads. Overall, EasyCluster2 is able to perform an effective detection of splicing isoforms, since it can refine exon-exon junctions and explore alternative splicing without known reference transcripts. Results in GFF3 format can be browsed in the UCSC Genome Browser. Therefore, EasyCluster2 is a powerful tool to generate reliable clusters for gene expression studies, facilitating the analysis also to researchers not skilled in bioinformatics.

## Background

Expressed sequence tags (ESTs) and full-length cDNAs (FL-cDNAs) are an invaluable source of evidence to infer reliable gene structures and discover potential alternative splicing events [1]. Their biological potential can be fully exploited through the clustering in which expressed sequences are linked to their specific gene loci of origin. To generate reliable gene-oriented clusters of ESTs, in presence of a complete or draft genome assembly, we developed EasyCluster that resulted the most accurate when compared to the state of the art software in this field [2-4]. Nowadays, thanks to technological advances, EST-like sequences can be produced by pyrosequencing using the Roche 454 platform. Indeed, this is a technology able to generate, through the GS FLX+ Titanium chemistry, sequence reads up to 1Kb long (http://www.454.com/) [5]. In addition, very long reads (more than 1Kb) can be currently obtained through the third generation of sequencers as the PacBio based on single molecule real-time (SMRT) technology (http://www.pacificbiosciences.com/) [6].

Handling huge amount of EST-like data is extremely useful to detect alternative isoforms, improve gene annotations or simply create gene-oriented clusters for expression studies. Since EST-like data provide a fragmented overview of their genomic loci of origin, transcript assembly may be an optimal solution to annotate user-produced sequences.

Since the advent of next generation sequencing technologies, however, no updated software to employ genome-based clustering of long transcriptome reads has been released. Indeed, as a novelty, we found the program [7], developed to group and assemble ESTs pre-aligned to a reference genome, and an improved wcd [8,9] release to cluster ESTs without genomic information. While wcd implements a new algorithm based on suffix arrays to handle huge amount of reads generated by high-throughput sequencers [8], RCDA has been conceived only for ESTs produced by the classical Sanger sequencing and, thus, never tested on long sequences produced by next generation technologies as those from Roche 454 [7].

To fill this gap and benefit from both genome assemblies and long transcriptome reads, we developed EasyCluster2 [10], a reimplementation of EasyCluster, that can now manage genome scale transcriptome data and produce reliable gene-oriented clusters from 454 reads, enabling the assembly of full-length transcripts and facilitating downstream analyses.

EasyCluster2 accepts read alignments in the standard GFF3 format (http://www.sequenceontology.org/gff3.shtml) generated by various mappers such as GMAP [11], refines the read clustering using information of shared splice sites, and resolves potential mapping errors at exon-exon junctions using dynamic programming.

The novel EasyCluster2 software can now handle unspliced reads (prominent in classical 454 data) and optimize the cluster definition with known gene annotations. A graph-based approach is used to assemble full-length transcripts belonging to a specific cluster, thus simplifying the investigation of post-transcriptional events as alternative splicing. Indeed, the AStalavista [12] program has been integrated in our tool allowing a quick way to explore alternative splicing without known reference transcripts.

In absence of large curated benchmarks in which the relationship between reads and genomic loci of origin is perfectly known, the reliability of EasyCluster2 has been assessed by simulated reads generated taking into account the Titanium Roche 454 chemistry. Same simulated datasets have been used to compare EasyCluster2 results to those obtained using other recent programs as RCDA [7] or clustering software not genome-based as wcd [8,9].

EasyCluster2 has been written in Java programming language and its graphical interface has the aim to simplify genome-level analyses to researchers not fully skilled in bioinformatics. The main executable and documentation is freely available at the Google code page: https://code.google.com/p/easycluster2.

## Methods

### Overview of EasyCluster2

The EasyCluster2 workflow is summarized in the following steps:

1 An individual alignment file in GFF3 format is provided as input and parsed in memory exploiting JAVA classes of a custom library. Then reads are grouped according to their 'exon' features included in the GFF3 file;

2 Initial clusters are generated by overlapping genomic coordinates;

3 Refined Clusters are then produced using to the biological criterion of splice site sharing;

4 Potential mapping errors are corrected by an *ad-hoc *re-alignment strategy;

5 Unspliced (intronless) and Mixed (multi-mapping) reads are included in relevant clusters using proper criteria;

6 Clusters can be merged to take into account the fragmented locus sequencing;

7 Known annotations (if available) can be exploited to improve clusters correctness;

8 Full-length transcripts are assembled from generated clusters by a graph-based procedure;

9 Alternative splicing events can be predicted using the embedded AStalavista module.

The software has been developed in Java programming language and tested on unix based machine equipped with 2 quadcore CPUs and 16GB of RAM.

### Cluster refinement in EasyCluster2

EasyCluster2 implements a novel strategy to refine the clustering procedure in order to mitigate the effect of alignment errors in regions surrounding splice sites.

The relative pseudocode is described in *Algorithm 1*.

### Transcript Assembly

A novelty of EasyCluster2 is the procedure to assemble full-length transcripts from reads allocated to specific clusters. The algorithm is based on the building of directed acyclic graphs and the pseudocode is described *Algorithm 2*.

**Algorithm 1 **Cluster refinement

1: **function **REFINECLUSTER()

2:    **for all ***exon *∈ *EST ***do**

3:      **if ***i*-exon is not the final exon of the EST **then**

4:        **take **the donor site of *exon_i _(donor_i_*)

5:        **take **the acceptor site of *exon_i_*_+1 _(*acceptor_i_*_+1_)

6:        **get **the *nucF asta *of the FASTA in an interval of 10 coordinates on the left of *donor_i _*and 10 coordinates on the right of *acceptor_i_+1*

7:        **create **new *nbDonor*[ ] and *nbAcceptor*[ ] which are classes composed by a coordinate, score and occurrence of that splicing site in the *P rof ileCluster*

8:        **for all ***j ∈ [−*5, 4] **do**

9:            *nbDonor[j *+ 5]*.coord ← donor_i _+ j *▷ *j *+ 5 is used for having array index from 0 to 9

10:            *nbAcceptor[j *+ 5]*.coord ← acceptor_i_*_+1 _+ *j*

11:      **end for**

12:      **for all ***j *∈ [0, 9] **do**

13:          **if ***nbDonor[j].coord *∈ *ProfileCluster*: **then**

14:            **get **the *nucGenomeInDonor *of the Genomic Sequence in an interval of 15 bases on the left of *nbDonor[j].coord *and 15 bases on the right of *nbDonor[j].coord*

15:            *nbDonor[j].score ← *SMITH-WATERMAN(*nucFasta, nucGenomeInDonor*) 16:            *nbDonor[j].occ ← ProfileCluster(nbDonor[j].coord).occ*

17:            **if ***nbAcceptor[j].coord ∈ ProfileCluster ***then**

18:              **get **the *nucGenomeInAcceptor *of the Genomic Sequence in an interval of 15 bases on the left of *nbAcceptor[j].coord *and 15 bases on the right of *nbAcceptor[j].coord*

19:              *nbAcceptor[j].score ← *SMITH-WATERMAN(*nucFasta, nucGenomeInAcceptor*)

20:              *nbAcceptor[j].occ ← ProfileCluster(nbAcceptor[j].coord).occ*

21:              **get ***maxScoreDonor *from *nbDonor[. . *. ]*.score *saving its index in the array *nbDonor[. . *. ]

22:              **get ***maxScoreAcceptor *from *nbAcceptor[. . *. ]*.score *saving its index in the array *nbAcceptor[. . *. ]

23:              **if ***maxScoreDonor > maxScoreAcceptor ***then**

24:                 **shift **the end part of the *exoni *on the start part of the *exon_i_*_+1 _with a displacement that depends on the coordinate in *nbDonor *which has the maximum score

25:            **end if**

26:            **if ***maxScoreDonor < maxScoreAcceptor ***then**

27:               **shift **the start part of the *exon_i_*_+1 _on the end part of the *exon_i _*with a displacement that depends on the coordinate in *nbAcceptor *which has the maximum score

28:            **end if**

29:            **if ***maxScoreDonor = maxScoreAcceptor ***then**

30:              **get ***occDonor *from *nbDonor[. . *. ]*.occ *using the saved index of the max donor score in the array *nbDonor[. . *. ]

31:              **get ***occAcceptor *from *nbAcceptor[. . *. ]*.occ *using the saved index of the max acceptor score in the array *nbAcceptor[. . *. ]

32:              **if ***occDonor > occAcceptor ***then**

33:                **shift **the end part of the *exon_i _*on the start part of the *exon_i_*_+1 _with a displacement that depends on the coordinate in *nbDonor *which has the maximum score

34:              **end if**

35:              **if ***occDonor < occAcceptor ***then**

36:                **shift **the start part of the *exon_i_*_+1_on the end part of the *exon_i _*with a displacement that depends on the coordinate in *nbAcceptor *which has the maximum score

37:              **end if**

38:              **if ***occDonor = occAcceptor ***then**

39:                No shifting

40:              **end if**

41:            **end if**

42:          **end if**

43:        **end if**                              ▷"coord" stands for "coordinate"

44:      **end for**                                ▷"occ" stands for "occurrence"

45:    **end if**                                  ▷"nuc" stands for "nucleotides"

46:  **end for**        ▷"nb" in *nbDonor *and *nbAcceptor *stands for "neighbourhood"

47: **end function**

**Algorithm 2 **Transcript Assembly

1:  **function **ASSEMBLETRANSCRIPTS()

2:      **generate ***StartList*[ ]                          ▷**if ***col_j _*∈ *matGraph *= 0

3:      **generate ***EndList*[ ]                        ▷**if ***rowi *∈ *matGraph *= 0

4:      **for all ***startNode_i _*∈ *StartList[. . *. ] **do**

5:          **create ***pathList*[ ]

6:          **add ***startNode_i _*in *pathList*[ ]

7:          **if ***startNode_i _*∈ *EndList[. . *. ] **then**

8:            **save ***pathList[StartNode_i_*]

9:          **else**

10:            **for all ***a(j) ∈ row(startNode_i_*) **do**

11:                **if ***a(j*) = 1 **then**

12:                  **add ***a(j*) IN *pathList[startNode_i_*]

13:                  RECURSIVEPATH(*a(j), pathList[. . *. ]*, coverMatGraph*)

14:                  **remove ***LAST ELEMENT *of *pathList[. . *. ]

15:                **end if**

16:            **end for**

17:        **end if**

18:      **end for**

19:  **end function**

20:

21:  **function **RECURSIVEPATH(*Node_j_, pathList[. . *. ]*, coverM atGraph*)

22:      **if ***Node_j _*∈ *EndList[. . *. ] **then**

23:        **save **pathList[. . . ]

24:      **else**

25:        **for ***a(i*) ∈ *row(Node_j_*) **do**

26:          **if ***a(i*) = 1 **then**

27:            **for ***index *∈ *pathList[. . *. ]-*lastIndex ***do**

28:              **if ***coverMatGraph[index, i*] = 1 **then**

29:                *coverMatGraph[index, i] ← *0

30:              **end if**

31:            **end for**

32:            **add ***a(i*) IN *pathList[. . *. ]

33:            RECURSIVEPATH(*a(i), pathList[. . *. ]*, coverMatGraph*)

34:            **remove ***LAST ELEMENT *of *pathList[. . *. ]

35:          **end if**

36:        **end for**

37:      **end if**

38:  **end function**

### Datasets

EasyCluster2 has been tested on two real datasets and simulated reads. As real dataset we used our human benchmark including 111 genes spread over almost all human chromosomes and 17,733 ESTs. Relationships between genes and ESTs were perfectly known. In addition, we used also the RCDA human dataset of 21,599 ESTs from chromosome 21, downloadable from http://140.226.190.96/iddrc/chr21/HSA21qGenesoftware.php.

Simulated Roche 454 reads were generated using the 454sim software [13] that allows the production of datasets under the 454 error model and different chemistries (GS20, GS-FLX, Titanium). For our purpose, we simulated 100,000 reads with the Titanium chemistry from 404 human transcripts (belonging to 213 genes) mapping on chromosome 21. The final dataset in FASTQ format was cleaned from adapters and low-quality sequences before any downstream use. The max read length was of 600 bp with a modal value in the range between 480 bp and 500 bp.

Tracing the origin of each simulated read, we created the benchmark to calculate accuracy metrics. In order to compare EasyCluster2 performances with those from RCDA and the oldest EasyCluster version working only on spliced reads, we generated a second simulated dataset including only interrupted reads.

Real and simulated datasets were mapped against the human genome (assembly hg19) by GMAP software, using the "-f 2" flag to get the output in gff3 format, suitable for EasyCluster2, and the "-Z" flag to produce a second output in compressed format needed for RCDA and the oldest EasyCluster version.

According to GMAP results, 46% of all reads appeared spliced, while 51% were unspliced with indeterminate orientation and 1% showed multiple mappings (mixed). Remaining reads (2%) were discarded by GMAP since too short for reliable alignments.

All datasets as well as clustering results are available at http://150.145.82.212/ernesto/easycluster2/easycluster2 datasets results.zip.

### Clustering software

RCDA program was downloaded from http://140.226.190.96/iddrc/chr21/HSA21qGenesoftware.php and run using default options as indicated in the provided documentation. Wcd program, version 0.6.3, was obtained from the following google code page http://code.google.com/p/wcdest/ and run using options -l 80 -T 60 -H 36 as recommended for 454 reads. The previous EasyCluster version was downloaded from http://150.145.82.212/ernesto/easycluster1/ and launched using default parameters.

In EasyCluster2 we set minimum read identity to 90% and minimum read coverage to 80%.

### Comparative evaluation of EasyCluster

Evaluation on benchmark and simulated datasets has been conducted calculating sensitivity and Jaccard index for each program outcome. Sensitivity is defined as $\frac{Tp}{Tp+Fn}$ (*Tp *= true positives, *Fn *= false negatives, hence *Tp + Fn *= all positives) and gives us an indication of the proportion of true ESTs that has been correctly placed in the correct reference clusters. The Jaccard index instead is defined as $\frac{Tp}{Tp+Fn+Fp}$ (*Fp *= false positives) and measures the similarity between predicted and reference clusters. Type I and Type II error rates have been calculated according to Wang *et al*. [14]. For each program outcome we calculated also the specificity that in combination with sensitivity, can provide a more concrete idea of clustering accuracy. All metrics were obtained using a custom python script [2].

### Implementation

EasyCluster is implemented in Java programming language and, thus, platform independent.

## Results

### General features of EasyCluster2

**Figure 1:**
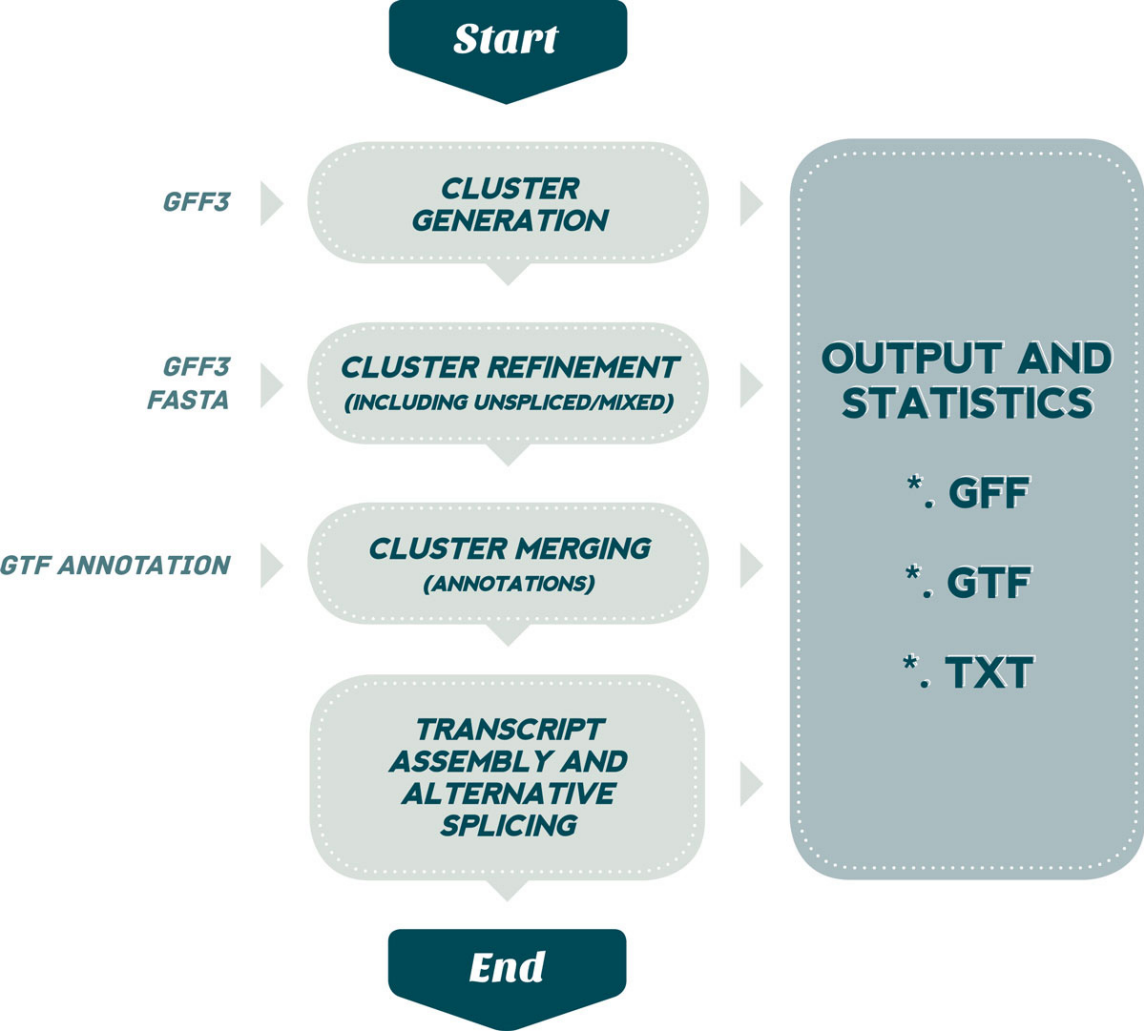
**Overview of EasyCluster2 workflow**. See main text for details.

The EasyCluster2 algorithm has been completely rebuilt and redesigned implementing new and unique features to improve the clustering process and facilitate the analysis to researchers without advanced skills in bioinformatics. The main Easy-Cluster2 procedure is depicted in Figure 1 and 1described below point by point.

#### GFF3 parsing and first clustering

In contrast with the previous version, EasyCluster2 accepts as input alignment files in thestandard GFF3 format and parse them in memory exploiting JAVA classes of a custom library. Along the parsing, read alignments are classified in Unique (occurring in only one genome location) and Mixed (mapping on multiple genome locations). In addition, reads are further divided into Spliced (including at least 1 intron) and Unspliced (intronless).

After the file traversing and read classification, Unique and Spliced sequences are grouped according to their 'exon' features leading to the creation of appropriated and dedicated data structures. Initial clusters are finally generated using overlapping genomic coordinates. The sorting of all read alignment coordinates is performed in advance to speed up the clustering procedure.

#### Second clustering and refinement

**Figure 2:**
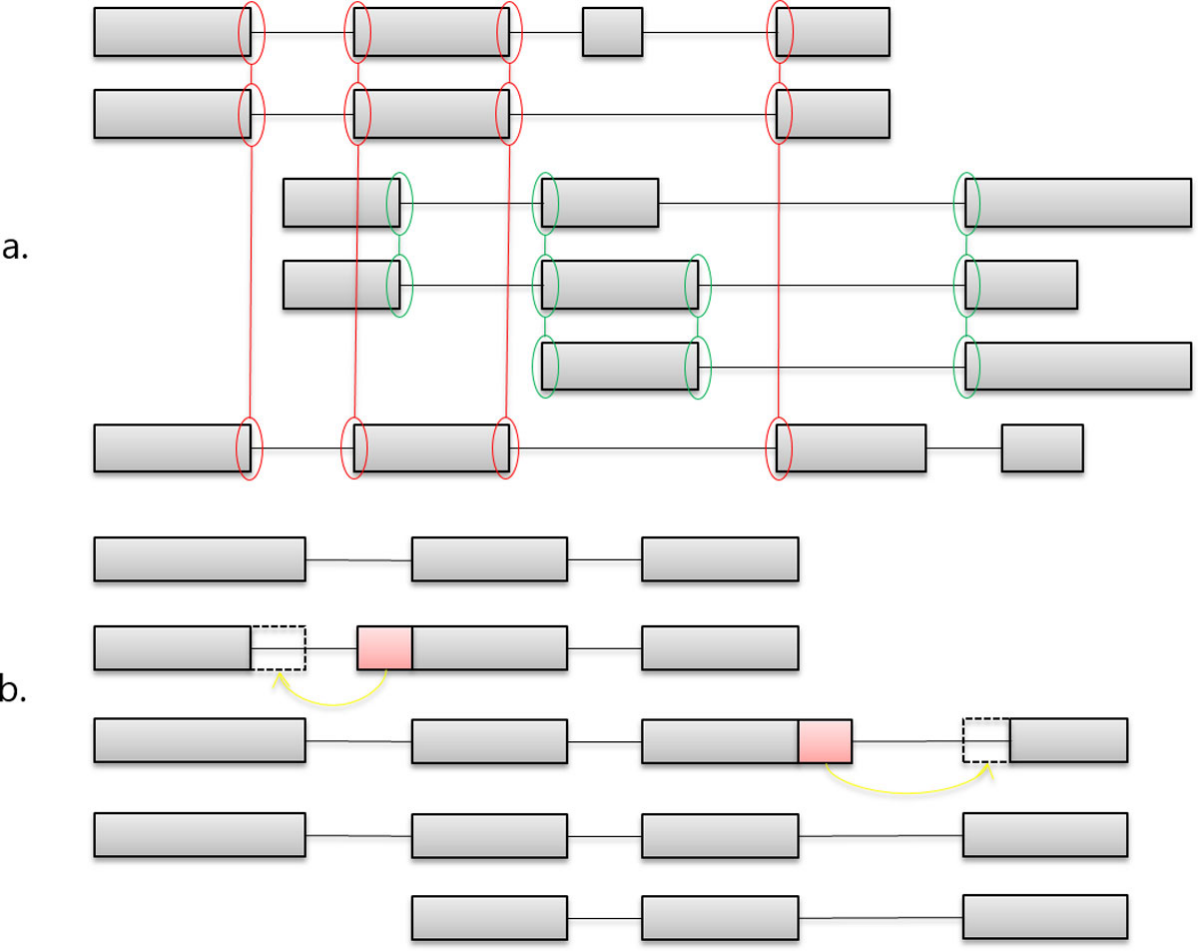
**Second Clustering and refinement**. a. Example of an initial cluster splits in two groups (red and green) by the splice site sharing criterion. b. Exon shifting after the refinement by Smith and Waterman algorithm in the region surrounding splicing sites. Squares are exons whereas lines are introns.

Initial clusters, defined also pseudo-Clusters, are then improved using the biological criterion of splice site sharing and, thus, only reads with at least one splice site in common are maintained in the same grouping (Figure 2a). Since the alignment of spliced reads onto the reference may not be optimal in regions surrounding splice sites, we introduce a refinement strategy based on an *ad-hoc *re-alignment procedure using the classical Smith-Waterman [15] algorithm.

From a computational point of view, for each cluster, we check the shift of a read substring (or rather a portion of its exons) considering all splicing sites as annotated in the cluster. In particular, the algorithm verifies a potential correspondence for all couples of donor (i-th exon) and acceptor (i+1-th exon) sites of each read (or exon); in other words, it checks if a specific coordinate of each read exon under analysis is already present in the cluster. This check is performed in a region of 10 nucleotides surrounding the splicing site, 5 upstream and 5 downstream, respectively. In case of a correspondence, the Smith-Waterman algorithm is used to verify the quality of the alignment onto the corresponding genomic region. The two exons under investigation are cut according to Smith-Waterman results and ready to be shifted to the previous or next exon as shown in Figure 2b.

#### Inclusion of Unspliced and Mixed reads

Unspliced reads are prominent in transcriptome sequencing experiments carried out by Roche 454 machines. For this reason, we implemented in EasyCluster2 a specific procedure to include Unspliced reads. In practice, these reads, for which the orientation is indeterminate, are included in already generated clusters if completely comprised in exonic regions. Alternatively, Smith-Waterman is applied to facilitate their allocation. Indeed, several Unspliced reads may be treated as Spliced reads due to misalignments of exon-exon junctions near the ends. All Unspliced reads not included in existent groupings, are released as independent clusters.

Mixed reads, instead, mapping on multiple genome locations, are optionally inserted in pre-constituted clusters according to a membership coefficient, calculated by the following formula:

$$mc=\frac{nESTmapSS}{totSScluster}$$

Where *totSScluster *is the number of splice sites in the examined Cluster and *nESTmapSS *is the number of examined Cluster reads that have the same Mixed read splice sites. Mixed reads are assigned to the cluster with the highest membership coefficient.

#### Cluster merging

EasyCluster2 implements now a procedure to merge adjacent clusters based on overlapping coordinates and strand orientation. In addition, if known annotations are available, they are exploited to link and merge groupings sharing splice sites, overlap of coordinates and strand orientation, improving the clusters correctness.

#### Transcript assembly

**Figure 3:**
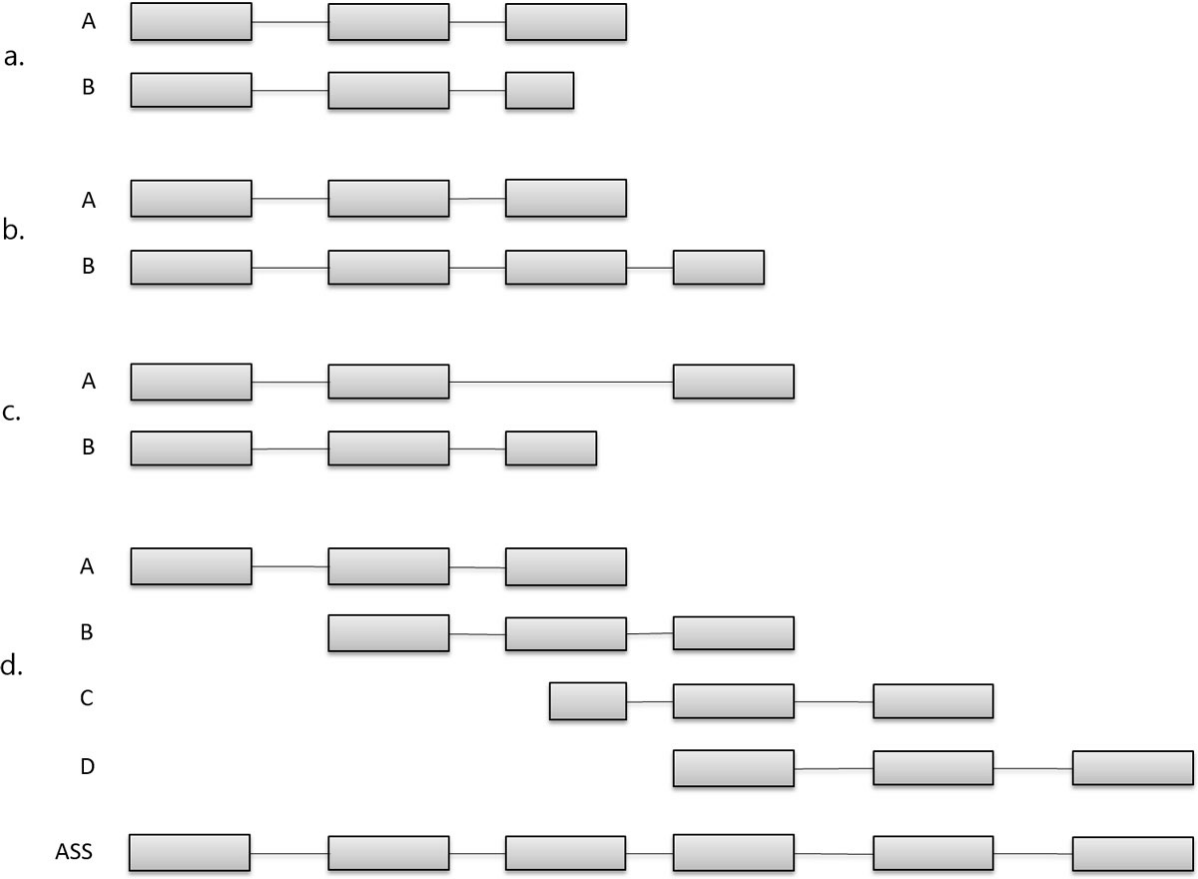
**Read assembly**. Inclusion and/or extension rules implemented in EasyCluster2: a. Inclusion; b. Extension; c. No relationship; d. Resulting full-length transcript.

As a novelty, EasyCluster2 is able to assembly clustered reads in full-length transcripts using a graph-based algorithm [16]. The main procedure is accomplished by solving the inclusion and/or extension between pairwise reads, verifying the sharing of splice sites (Figure 3a-c). Indeed, given an ordered pair of reads, we may have at least three scenarios:

1 Inclusion, if both reads share all splice sites and the size of the second read is smaller than the size of the first one;

2 Extension, if both reads share splice sites and the size of the second reads is greater than the size of the first one;

3 No relationship, if both reads have discordant splice sites.

According to above rules, EasyCluster2 builds a directed acyclic graph and performs transcript assembly (Figure 3d ).

#### Alternative splicing

The prediction of alternative splicing is a basic step once full-transcripts have been generated per each cluster. Although many programs tend to predict alternative splicing events defining a reference transcript and then valuating differences in the pattern of splice sites, EasyCluster2 takes a different approach. Indeed, it implements the AStalavista [12] program in which splice site inconsistencies and, thus, alternative splicing events are detected by looking at genomic coordinates without any reference transcript. This is an optimal solution in the case of gene annotations are not well known and no reference transcripts can be defined unambiguously.

### Assessment of EasyCluster2 performance

EasyCluster2 accuracy was initially checked on our standard benchmark dataset comprising 111 human genes spread over almost all human chromosomes and 17,733 ESTs (including RefSeqs and alternative transcripts) [2]. This benchmark also includes cases of overlapping and nested genes and the relationship between each gene locus and corresponding ESTs of origin is perfectly known. In this limited dataset, Easycluster2 correctly predicted all 111 clusters outperforming the previous Easy-cluster implementation in which only 109 were exactly detected [2].

We also tested our software on a second human dataset including 21,599 transcripts from chromosome 21. This dataset was used to compare the performance of a recently released program named RCDA with the first version of EasyCluster [7]. RCDA predicted 379 clusters while EasyCluster produced 389 groupings in 90 min. In contrast, Easycluster2 generated 354 clusters compatibles with current annotations in UCSC in less than 10 min using default parameter or less than 30 min activating the exon refinement on a laptop computer. Full-length transcripts were reconstructed in minutes and appeared consistent with RefSeq annotations included in the dataset. The consistency was estimated looking at shared introns since upstream and downstream transcript regions may differ.

Finally we assessed the performance of EasyCluster2 using a dataset of human reads simulated according to the Roche 454 error model and Titanium chemistry. Simulated reads were generated using known human chromosome 21 RefSeq annotations from the UCSC genome browser. The overall performance was evaluated in terms of sensitivity, specificity and Jaccard Index (see Materials and Methods for further details). In addition, we generated also a dataset including only spliced reads to compare results to those from RCDA and the oldest release of EasyCluster. Both datasets were also used to assess the read clustering by the novel wcd program that does not take into account the human genome assembly.

In case of RCDA, the previous EasyCluster release and EasyCluster2, simulated reads were aligned onto the reference human genome (assembly hg19) using GMAP software [11].

Main results on both datasets are reported in Table 1. When only spliced reads are taken into account, EasyCluster2 outperforms other programs in terms of sensitivity and specificity. Very low values of type I (mis-separation error) and type II (mis-joining error) errors have also been registered for EasyCluster2. Concerning the full dataset including spliced as well as unspliced reads, EasyCluster2 performs better than wcd, even though this comparison is a bit biased because wcd is an *ab initio *clustering tool and, thus, does not require any genomic information during the grouping of reads. Using the full dataset, we registered a high type I error for EasyCluster2 (Table 1 ), meaning that several clusters should be grouped together. This is an expected behaviour for Roche 454 datasets in which the relatively low throughput (as well as the heterogeneous read length) prevents the complete coverage of expressed genomic loci. In contrast, type II error for EasyCluster2 is very low suggesting that only a few reads are incorrectly assigned to the right cluster.

**Table 1:** Software assessment. For each program we report the sensitivity (Sn), the specificity (Sp), the Jaccard Index [22], the type I error (EI) and the type II (EII) error. EasyCluster1 refers to the oldest EasyCluster version, whereas EasyCluster2+Ann is the new implementation in which final clusters are refined by known annotations.

Dataset including only spliced reads					
Program	Sn	Sp	JI	EI	EII

EasyCluster1	0,732	0,975	0,732	0,214	0

RCDA	0,783	0,969	0,770	0,147	0,006
wcd	0,754	0,913	0,708	0,178	0,030

EasyCluster2	0,825	0,950	0,805	0,122	0,024

Full Dataset (spliced + unspliced reads)					

Program	Sn	Sp	JI	EI	EII

wcd	0,520	0,850	0,377	0,335	0,037

EasyCluster2	0,675	0,930	0,637	0,490	0,047

EasyCluster2+Ann	0,828	0,905	0,807	0,019	0,037

Roche 454 tends to produce many unspliced reads (sometimes more than 50% of all reads as other next generation sequencing technologies) that are generally discarded during the genome-based clustering. EasyCluster2 provides now the opportunity to employ all categories (spliced, unspliced and mixed) of reads from a 454 experiment and this feature represents the most desirable scenario for a researcher.

As shown in Table 1 (see row EasyCluster2+Ann), EasyCluster2 type I error sensibly decreases providing known annotations. Indeed, gene annotations are extremely useful to link compatible clusters in terms of overlapping coordinates or splice sites sharing, improving the overall clustering procedure.

## Discussion

While DNA microarray data are used to identify genes which could be considered prognostic markers [17], to predict protein interactions [18] and to discover molecular pattern [19], ESTs are essential for gene discovery, gene mapping, genome annotation, SNP discovery, alternative splicing detection and RNA editing prediction.

ESTs are essential for gene discovery, gene mapping, genome annotation, Single Nucleotide Polymorphism (SNP) discovery, alternative splicing detection and RNA editing prediction. With the advent of next generation sequencing technologies, huge amount of EST-like sequences can be produced at relatively low cost, enabling more detailed transcriptomic as well as genomic investigations. Although several programs have been developed to analyse short reads from complete transcriptomes [20], very few tools are available to handle long reads produced by sequencers as the Roche 454 or PacBio. Thanks to the Titanium chemistry, the Roche 454 sequencer is able to generate reads longer than 600 bp (and in theory up to 1Kb) that are in the same range of ESTs produced by the standard Sanger methodology. Although Roche 454 has not a very high-throughput, generating up to one million reads per run, the analysis of resulting data is yet a challenging task. Indeed, at least the 50% of transcriptomic reads by Roche 454 experiments are unspliced and not strand oriented. Therefore, after the mapping onto the corresponding reference genome, they have an indeterminate orientation.

Existing programs to cluster EST-like sequences using genome alignments, as EasyCluster or RCDA, tends to exclude unspliced reads, leaving precious biological information. On the other hand, clustering programs based on EST sequences only, as wcd, can employ all reads even though resulting clusters are quite questionable. In absence of additional genomic evidence, similarity-based methods suffer from notable limitations. Indeed, ESTs/reads from paralogous genes or from nested and overlapping genes may not be correctly clustered. Few years ago, we developed EasyCluster, a genome-based EST clustering tool able to employ EST to genome alignments to reconstruct reliable gene-oriented clusters. Although it appeared more accurate than state of the art software, EasyCluster cannot handle long reads from next generation sequencing technologies and benefit from biological evidence of unspliced reads [2]. For this reason we developed EasyCluster2 [10] in which the algorithm implemented in EasyCluster has been completely redesigned to take into account an increased number of reads and dedicated procedures to improve the clustering process including also unspliced and mixed reads. Since EasyCluster2 employs progressive read alignments onto the reference genome, potential errors in regions surrounding splice sites may alter the quality of subsequent clusters. To solve this issue, EasyCluster2 implements an *ad hoc *procedure to refine alignments near splice sites using dynamic programming.

To demonstrate the reliability of EasyCluster2 we compared our software with two recently released programs to cluster ESTs, RCDA [7] based on EST to genome alignments and wcd [8], an improved similarity-based tool. In order to make unbiased the comparison, we generated simulated Roche 454 reads in which the relationship between reads and genomic locus of origin is perfectly known. Limiting the analyses to spliced reads, EasyCluster2 resulted more accurate than RCDA and wcd in terms of sensitivity and Jaccard index (Table 1 ). When all reads were taken into account, EasyCluster2 outperformed wcd (Table 1 ). Very interestingly we obtained a very low type II error due to mis-joining, meaning that only a reduced fraction of reads was allocated to wrong clusters. In absence of gene annotations, we registered a high type I error due to mis-separation because many genomic loci appeared not completely covered by reads leaving to more than one expected cluster. However, this type of error can be mitigated including additional biological evidence (more reads) or reliable annotations (Table 1).

As a novelty, EasyCluster2 can assemble cluster of reads in full-length transcripts and predict alternative splicing events thanks to the embedded AStalavista tool.

On the whole, EasyCluster2 is a novel tool ready to handle long reads from next generation sequencing, providing reliable clusters that can be used as strong evidence sources to improve gene-finding procedures or explore complex transcriptomes as well as post-transcriptional events herein as alternative splicing and RNA editing. In addition, genome-based clusters can be employed for gene expression studies or identify tissue specific transcript variants.

## Conclusions

EasyCluster2 is a reimplementation of EasyCluster software devoted to the generation of gene-oriented clusters by massive transcriptome reads. Our software is written in Java language and implements different novelties including a procedure to mitigate mapping errors at splice sites and an *ad hoc *solution to assemble full-length transcripts per cluster. In addition, EasyCluster2 can now predict alternative splicing events thanks to the embedded AStalavista module.

Given the explosion of next generation sequencing and the concomitant increment of read lengths, we think that a tool as EasyCluster2 may be extremely useful for large-scale transcriptome experiments from 454 Roche or PacBio sequencers enabling complex genomic analyses to researchers not fully skilled in bioinformatics. Indeed, results demonstrate the high accuracy of EasyCluster2 in producing effective clusters as well as reliable full-length transcripts.

As future plans, we are working to extend EasyCluster2 to datasets from the Illumina platform in order to take into account paired-end reads and huge amount of reads. In addition, we are also planning to include in EasyCluster2 a procedure to accept in input alignments in the standard SAM/BAM format [21].

## Availability and requirements

Project name: *EasyCluster2*

Project home page: https://code.google.com/p/easycluster2

Operating system: Platform Independent

Programming language: *Java*

License: *Apache License 2.0*

Restrictions: none

## Competing interests

The authors declare that they have no competing interests.

## Authors' contributions

EP and GP conceived the study. EP and VB supervised the development. EP, VB, NP, EIG and FS designed the software. NP, EIG and FS developed the software. EP, VB, NP, EIG and FS drafted the manuscript. DS tested the software on real and simulated dataset. All authors read and approved the final manuscript.

## Declarations

Publication charges for this article have been funded by the Flagship Project Epigen at the National Research Council (CNR - Italy).

This article has been published as part of *BMC Bioinformatics *Volume 15 Supplement 15, 2014: Proceedings of the 2013 International Conference on Intelligent Computing (ICIC 2013). The full contents of the supplement are available online at http://www.biomedcentral.com/bmcbioinformatics/supplements/15/S15.
